# Bayesian Estimation of Generalized Log-Linear Poisson Item Response Models for Fluency Scores Using brms and Stan

**DOI:** 10.3390/jintelligence13030026

**Published:** 2025-02-23

**Authors:** Nils Myszkowski, Martin Storme

**Affiliations:** 1 Department of Psychology, Pace University, New York, NY 10004, USA; 2 IESEG School of Management, Université de Lille, CNRS, UMR 9221-LEM-Lille Economie Management, 59000 Lille, France; m.storme@ieseg.fr

**Keywords:** divergent thinking, fluency, creativity, item response theory, psychometrics, Bayesian estimation

## Abstract

Divergent thinking tests are popular instruments to measure a person’s creativity. They often involve scoring fluency, which refers to the count of ideas generated in response to a prompt. The two-parameter Poisson counts model (2PPCM), a generalization of the Rasch Poisson counts model (RPCM) that includes discrimination parameters, has been proposed as a useful approach to analyze fluency scores in creativity tasks, but its estimation was presented in the context of generalized structural equation modeling (GSEM) commercial software (e.g., Mplus). Here, we show how the 2PPCM (and RPCM) can be estimated in a Bayesian multilevel regression framework and interpreted using the R package brms, which provides an interface for the Stan programming language. We illustrate this using an example dataset, which contains fluency scores for three tasks and 202 participants. We discuss model specification, estimation, convergence, fit and comparisons. Furthermore, we provide instructions on plotting item response functions, comparing models, calculating overdispersion and reliability, as well as extracting factor scores.

## 1. Introduction

A number of psychological measurements produce item scores that consist of a count of events in a fixed amount of time. For example, verbal fluency tasks may ask participants to generate as many words as possible that start with a specific letter. Some measurement procedures may involve examinees making multiple attempts (e.g., to solve math problems, to read words) in a given amount of time; we may then count the number of successful (or unsuccessful) attempts. In the field of creativity research, divergent thinking tasks, which generally ask examinees to produce as many creative ideas as possible in response to a given prompt, are often scored (at least partly) based on fluency, which refers to the count of ideas generated in response to the prompt. For example, divergent thinking tasks may ask participants to generate as many uses as possible for an object (e.g., a brick) (Torrance 2008) or to find as many different explanations for a social situation (Preckel et al. 2011). Beyond the measurement of divergent thinking ability in general, the same kind of test has been adapted to more specific domains. For example, one may attempt to measure managerial divergent thinking by asking examinees to find as many ideas as possible to strengthen cohesion within a team (Myszkowski et al. 2015), or to measure engineering design divergent thinking by asking examinees to find as many design ideas for a given problem (e.g., design an object that produces sounds) (Charyton and Merrill 2009).

The tasks given as examples here therefore produce scores which are discrete and have a lower bound at 0, which generally mean that they are right-skewed when low counts are expected. Also, when higher scores are expected, they tend to be more variable, which implies a mean–variance relationship. These characteristics make them unsuitable for traditional factor analysis, which generally assumes continuous, normally distributed variables with fixed variance. Consequently, item response models that assume scores to follow different probability distributions, such as the Poisson distribution, have been suggested as more appropriate for divergent thinking tasks (Forthmann et al. 2016; Myszkowski 2024; Myszkowski and Storme 2021), as well as for other tasks that produce counts (Doebler and Holling 2016; Jansen 1986; Meredith 1971; Rasch 1960; Spray 1990).

### 1.1. The Rasch Poisson Counts Model

The original item response theory model developed for count item responses is the Rasch Poisson counts model (RPCM; Rasch 1960). It defines the fluency score $X_{ij}$ for person *i* (where i=1,…,n) on item *j* (where j=1,…,m) as following a Poisson distribution of rate $\lambda_{ij}$:

(1) $$X_{ij}∼\mathrm{Poisson}\left(\lambda_{ij}\right).$$

In the Poisson distribution, the rate parameter $\lambda_{ij}$ is equal to both the mean and the variance of the distribution. This relation of equality between the mean and the variance is a characteristic of the Poisson distribution, which is known as equidispersion. In the RPCM, the rate parameter is modeled as a function of an item easiness parameter $b_{j}$, a person ability parameter $\theta_{i}$ and a general discrimination parameter *a*, such as the following:

(2) $$\lambda_{ij}=e^{b_{j}+a\theta_{i}}.$$

The use of the exponential function as the inverse link defines this model as a log-linear model (Doebler et al. 2014; Mellenbergh 1994; Myszkowski 2024), although this has also been referred to as a multiplicative model (e.g., Jansen 1986). The absence of other item scores in the response function reflects that, as is usual in measurement models, it is assumed that scores are conditionally independent, meaning that they are not related over and beyond $\theta_{i}$. This assumption, commonly referred to in the IRT literature as local independence (Chen and Thissen 1997), can be violated in various tests, including divergent thinking tests. For example, Myszkowski and Storme (2021) found that this was the case when there are similarities between certain prompts in a test (e.g., all tasks that are alternate use tasks in a broader set of tasks) that are not accounted for (using, for example, a bifactor/testlet model).

### 1.2. The Need for Item-Specific Discrimination Parameters

In tasks like verbal fluency tasks, one may expect (or voluntarily assume) that the number of responses only depends on a person’s latent ability and the difficulty of the prompt. For example, we would expect that generating words starting with the letters “A” or “Z” are prompts that vary in difficulty, but not in how strongly they are related to the underlying ability of verbal fluency. Certainly, in such contexts, the RPCM may be a reasonable model.

But, in other tasks, such as divergent thinking tasks, as illustrated by Myszkowski and Storme (2021), it may be suspected that different items could tap into (for example) different domains of expertise or cognitive abilities. For example, generating alternate uses of a knife could be related to expertise in cooking, while generating uses of a brick could be related to expertise in construction. In addition, different items could reflect more or less the underlying ability of interest due to nuisance factors (e.g., typing/writing speed, social inhibition). For example, high social inhibition may prevent certain ideas to be produced for alternate uses of certain objects (e.g., a knife) as opposed to others (e.g., a tire). As a consequence, because they may be confounded by different item-specific factors, it has been suggested that measurement models applied to divergent thinking fluency scores should allow for the possibility that, even accounting for item difficulty, different items may be more or less sensitive to variation in the underlying ability (latent fluency).

### 1.3. The 2-Parameter Poisson Counts Model (2PPCM)

The 2-parameter Poisson counts model (2PPCM Myszkowski and Storme 2021) was therefore proposed as a generalization of the RPCM, which allows for item-specific discrimination parameters. In the original paper, a re-analysis of a dataset containing various divergent thinking item responses (Silvia 2008a, 2008b, 2008c; Silvia et al. 2008) indicated that fluency tests may be more accurately modeled using the 2PPCM than the RPCM, even though, at this stage, further research is needed to confirm whether the 2PPCM is generally more accurate than the RPCM in divergent thinking tasks.

Like the Rasch Poisson counts model, the 2-parameter Poisson counts model (2PPCM) defines the fluency score for a participant *i* on item *j* as follows:

(3) $$X_{ij}∼\mathrm{Poisson}\left(\lambda_{ij}\right),$$

but, unlike the RCPM, the rate distributional parameter is modeled using a second item parameter, which is a discrimination parameter $a_{j}$:

(4) $$\lambda_{ij}=e^{b_{j}+a_{j}\theta_{i}}.$$

It can be seen that 2PPCM is a generalization of the Rasch Poisson counts model (RPCM), which is obtained when all $a_{j}$ are constrained to be equal.

In the psychometric literature, the former parametrization is typically referred to as a slope–intercept parametrization. Alternately, we may reparametrize the model in a more traditional IRT parametrization, which focuses on the distance between difficulty and ability parameters $\delta_{j}-\theta_{i}$:

(5) $$\lambda_{ij}=e^{\alpha_{j}\left(\delta_{j}-\theta_{i}\right)}.$$

A similar reparametrization can be used for the RPCM. Because it is more convenient to use a slope–intercept parametrization when using a regression framework, we will use it in the following sections. In other words, the rest of this paper uses Equation (4) as the model equation for the 2PPCM, not Equation (5).

### 1.4. Model Identification

Model identification for the 2PPCM obeys similar constraints and solutions as any other item response models with variable slope parameters. More specifically, in order to identify the 2PPCM, we generally choose to fix the latent variance (to 1, typically), which is often referred to as the variance standardization method. Because it has practical advantages when studying psychometric instruments, such as easily producing person location estimates on a standard normal (i.e., *z*) scale, facilitating the interpretation of discrimination parameters for all items, or easily interpreting person covariate estimates in latent regression models (by standardizing the covariate, we directly obtain standardized regression coefficients), this is often the preferred method in IRT. Although it may not be preferred for all modeling scenarios (e.g., using anchor items in differential item functioning), it is the default in popular IRT packages like ltm (Rizopoulos 2006) and mirt (Chalmers 2012).

An alternative which is more frequently the default in structural equation modeling software—such as lavaan (Rosseel 2012)—is to fix one of the slope parameters $a_{j}$ (to 1, typically), while the variance of $\theta_{i}$ is freely estimated. This is known as the marker method. We can note that, in the RPCM, fixing any $a_{j}$ to 1 implies that we are fixing all slopes to 1 (since the slopes are constrained to be equal).

### 1.5. Software

The 2PPCM can be estimated using generalized structural equation modeling (GSEM) software that accept Poisson distributions and logarithm link functions, such as Mplus or Stata, and was originally presented using this type of environment (Myszkowski and Storme 2021). Unfortunately, to the best of our knowledge, there is no open-source GSEM package that supports Poisson distributions and logarithm link functions. An alternative is to estimate models using generalized linear mixed models (GLMMs) software, such as the R package lme4 (4.4.2) (Bates et al. 2015), which can be used to estimate the RPCM (see Baghaei and Doebler 2019, for a tutorial). However, GLMM software do not allow for the estimation of the 2PPCM, because they do not allow for item-specific discrimination parameters. A notable exception is the R package PLMixed (Jeon and Rockwood 2018), which extends lme4 to allow for discrimination parameters like in the 2PPCM.

We shall note that different software environments impose constraints on the identification method. While GSEM software (e.g., Mplus) typically allow both identification methods, frequentist multilevel estimation software often do not. For example, lme4 (Bates et al. 2015)—which can be used for the RPCM (Baghaei and Doebler 2019)—and its extension for factor structures PLMixed (Jeon and Rockwood 2018) do not allow for fixing the latent variance and therefore limit the identification to the marker method.

Because recent research has highlighted that Bayesian estimation through Stan (Carpenter et al. 2017) and the brms package (Bürkner 2017) allows a number of interesting flexibilities for item response theory analysis (Bürkner 2020, 2021), we propose to explore its capacities in the analysis of count data using Poisson models. In other words, in this paper, we discuss the use of brms and Stan to estimate the 2PPCM (and, by extension, the RPCM) on a divergent thinking dataset.

### 1.6. Bayesian Item Response Theory Using brms and Stan

Although more extensive overviews of Bayesian item response theory (IRT) models using Stan and brms have been published (Bürkner 2020, 2021), we will provide a brief overview of the approach here. brms is an R package that provides an interface for R users for Stan (Carpenter et al. 2017), which is a probabilistic programming language that allows for the estimation of various Bayesian models using Hamiltonian Monte Carlo (HMC) sampling (Neal 2011), a type of Markov Chain Monte Carlo (MCMC) sampling. brms allows for the use of a regression-type syntax similar to the one used in the popular frequentist package lme4 (Bates et al. 2015) (and more generally, to the formula argument in R functions), which makes it substantially easier to use for researchers familiar with this package compared to using Stan directly. Many different response distributions can be used with various link functions, including, respectively, the Poisson distribution and the logarithm link function, which are, of course, particularly relevant here.

In a Bayesian framework, we specify a prior distribution for the parameters of the model, which is then updated using the data to obtain a posterior distribution. This posterior distribution is then used to make inferences about the parameters of the model. The principal downside of Bayesian estimation is that it is computationally intensive, and can be slow for complex models with many parameters. However, recent advances in Hamiltonian Monte Carlo sampling have made Bayesian estimation more efficient and accessible.

In the context of IRT, Bayesian estimation offers several advantages. One key benefit is the ability to set prior distributions on parameters, which can serve multiple purposes. First, priors can incorporate prior knowledge or beliefs about item (or person) parameters, which may be valuable when data are limited or when drawing on previous research findings. Second, priors can aid in convergence by stabilizing estimates, especially in complex models with many parameters. Finally, priors can act as a form of regularization, shrinking parameter estimates toward more “reasonable” values, thereby mitigating issues like over-fitting. Moreover, Bayesian IRT enables a straightforward interpretation of uncertainty through posterior distributions, providing richer information than point estimates alone. Rather than focusing solely on parameter estimates, researchers can examine the full distribution of possible values for each parameter, which helps in understanding the precision of estimates and the credibility of model-based inferences. A more thorough discussion of the benefits of Bayesian IRT in general can be found in Fox (2010), while a more specific discussion of Bayesian estimation in brms in IRT can be found in Bürkner (2021). In addition, an example tutorial for estimating binary logistic response models can be found in Bürkner (2020).

### 1.7. Aim of the Present Paper

In this paper, we show how the 2PPCM can be estimated in a Bayesian multilevel regression framework and interpreted using brms. We will illustrate this using the example dataset provided for the special issue, which contains fluency scores for 3 divergent thinking tasks (i.e., 3 items) and 202 respondents. We will discuss model specification, estimation, convergence, fit and comparisons. Furthermore, we will provide instructions on plotting item response functions and item information functions, comparing models, diagnosing model fit, checking equidispersion, calculating reliability, and extracting factor scores. Although we limit ourselves to the core components of IRT analysis and do not address all possible topics (e.g., differential item functioning, explanatory IRT, other counts distributions), we hope that this paper will provide a useful starting point for researchers interested in using Bayesian estimation for IRT models in the context of divergent thinking tasks.

In the paper itself, we present the most critical aspects of the code (e.g., the code will not present how to customize plots). This is both to keep the paper (relatively) concise and to minimize the risk of it becoming obsolete as packages evolve. The full code used, including the data, is available on the Open Science Framework (OSF) at https://osf.io/z8r7v/ (14 February 2025). Because it may evolve and depends on operating system characteristics, we defer to online tutorials for the installation of brms and Stan. Currently, links to install the brms package and its necessary components can be found at https://github.com/paul-buerkner/brms (accessed on 14 February 2025).

## 2. Model Estimation

### 2.1. Data Preparation

We used the dataset provided for the special issue, which has been presented in previous research (Forthmann et al. 2019; Forthmann and Doebler 2022; Forthmann et al. 2020). The participants were prompted to generate as many uses as possible for a rope (item 1), a paperclip (item 2) and a garbage bag (item 3); for the purpose of this paper, we analyzed only the part that contains the fluency scores. For convenience, the OSF repository contains the subset of the dataset that was used in the analysis. The item responses were pivoted to a long format (i.e., one row per participant per item). The data used throughout are called data_long, and contain a variable with the subject identifier (Person), the item identifier (Item), and the fluency score (Score). All cases have fluency scores for all items, except for one person who only has a score to the paperclip item; this case was kept in analysis. To note, a wide format of the data (data_wide) is also provided in the OSF repository, as it was used to produce Mplus analysis used as the benchmark.

### 2.2. Loading Libraries

We first load the brms library with


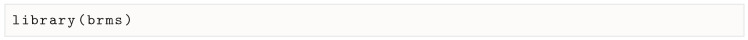


Throughout, we will also use the dplyr library (Wickham et al. 2023), notably to filter data frames conveniently:


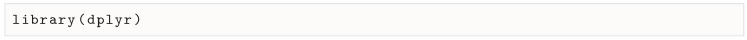


### 2.3. Model Specification

The distributional assumption and the item response function of the 2PPCM, respectively, presented in Equations (3) and (4), can be specified in brms using the bf() function:


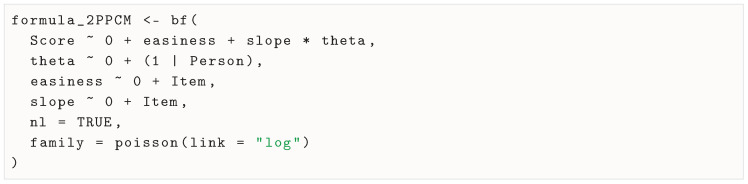


The formula is defined in a similar way to the formula used in the lme4 package, with the response variable on the left side of the tilde and the predictors on the right side. In the first part of the formula, we define the outcome variable (Score) as a function of the item and person parameters with Score ∼ 0 + easiness + slope * theta, which is a direct equivalent of the item response function of the 2PPCM as defined in Equation (4), except that it is not exponentiated, because the logarithm is later defined as a link function. The intercept is omitted with the 0 +, as is typical in IRT (this allows for item parameter estimates to correspond to item locations). To note, the two item parameters and person parameter in this first part of the formula are not observed in the dataset, but we define them as being predicted by variables in the dataset in the next lines of code.

The latent variable θ is defined using theta ∼ 0 + (1 | Person), which defines it as a random intercept (i.e., location) by grouping the variable (the variable Person in the dataset. Again, 0 + is used, which implies that the population mean of θ is set to 0. Both the easiness and the slope parameters are defined using fixed effects of the Item variable, using, respectively, easiness ∼ 0 + Item and slope ∼ 0 + Item. Alternatively, they may be defined as random effects, although it is not common practice in IRT, but see Bürkner (2021) for an example of how to do this. Finally, we declare the model as nonlinear with nl = TRUE and specify that the outcome variable follows a Poisson distribution with a log link function using family = poisson(link = "log"). We summarize the arguments of the bf() function in Table 1.

To note, although there are less verbose ways to specify it, it is easy to reuse the formula for the RPCM by fixing the slope parameter to be constant across items with slope ∼ 1:


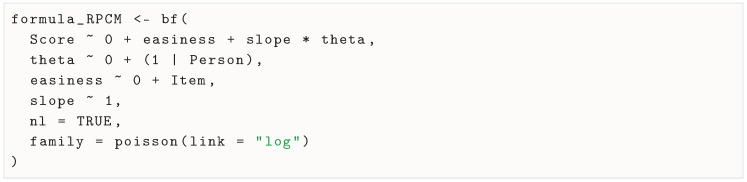


### 2.4. Setting Prior Distributions

Before setting priors, it is advisable to inspect what default priors are used by brms for the model. This can be carried out using


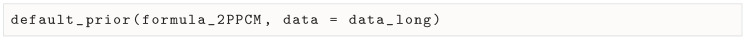


Currently, non-informative flat priors are used by default for all item parameters (easiness and slopes), and truncated Student’s *t* priors with a lower bound at 0, 3 degrees of freedom and a scale of 2.5 are used for the standard deviation of the Person random effect (i.e., the standard deviation of θ). Because we need to identify the model and have chosen to do so using the variance standardization method, it is necessary to fix the standard deviation of θ to a constant (1) instead. We can do this using the prior() function:


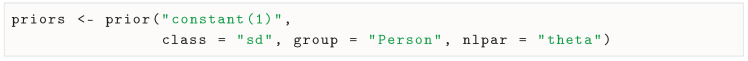


Priors may be set on easiness and slope parameters to stabilize the model, incorporate prior knowledge or regularize parameters. When doing this, one must keep in mind the logarithmic link function, which implies that the scale of the priors is not the same as for linear models. More specifically, the exponentiated easiness parameter corresponds to the expected count for someone of average ability ($\theta_{i}=0$), and the exponentiated slope parameter corresponds to the multiplicative effect of a one-standard-deviation increase in $\theta_{i}$ on the expected count. In our experimentations, we found that using flat priors on the easiness parameters was not problematic for model estimation, but that informative priors on the slope parameters were necessary to stabilize the model in some cases, including in the dataset at hand. Although it was only necessary for slope parameters, we discuss priors for both item parameters.

#### 2.4.1. Informative Priors for Easiness Parameters

Since the exponentiated easiness parameter corresponds to the expected count for someone of average ability, to help the model converge, a useful prior for easiness would be a distribution that spans (on a log scale) the (hypothesized or observed) range of observed counts. As a weakly informative prior, for example here, since scores range between 1 and 23 (thus a range of 22), we could use a normal distribution, truncated at 0, with a mean of $\log\left(1+\frac{22}{2}\right)(\approx$2.48) (which corresponds to the log of the midpoint) and a standard deviation corresponding to (the log of) half the range (log(11)≈2.40). We can add this new prior with the following:


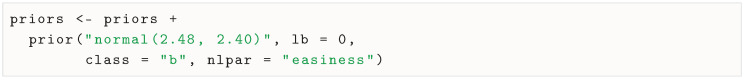


The first line of the prior() function generates the string that defines the distribution (lb = 0 is used to truncate the distribution), while the rest describes which prior is being modified.

#### 2.4.2. Informative Priors for Slope Parameters

We propose as a weakly informative prior for the slope parameters a normal distribution (truncated to positive values) with a mean of 0 and a standard deviation that corresponds to some expectation for the (log) maximum slope. In our example, we can hypothesize that a standard deviation of θ would at most quadruple the number of ideas in this context (i.e., a 4-fold increase), so we set the standard deviation of the prior to log(4) (≈1.39). We can add this new prior with the following:


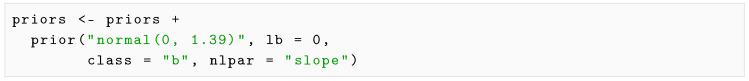


To note, although it is not generally a good idea to set priors based on the data because it risks over-fitting, a possible alternative, although it was not carried out here, could be to first estimate an RPCM on the data to obtain a common estimate for the discrimination parameter *a*, and to use this estimate as the mean of the prior for the slope parameters $a_{j}$ in the 2PPCM.

#### 2.4.3. Notes on Using the Marker Method of Identification

In this paper, we chose to use the variance standardization method of identification, which is the most common method in IRT. To use the marker method of identification, one would need to fix one of the slope parameters (usually the first) to a constant (usually 1).


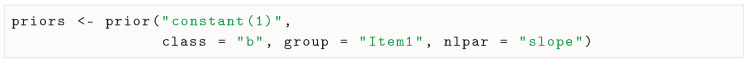


Because the first item slope is fixed to 1, we expect slopes to be close to 1 for all the other items, as long as the test is homogeneous (i.e., similar slopes for all items). In this case, because the items are all alternate uses tasks taken in similar conditions, we expect it to be the case. We can thus use as a weakly informative prior for the other slope parameters a normal distribution with a mean of 1 and a standard deviation that corresponds to some expectation for the range of slopes. Larger ranges shall be expected when items are more heterogeneous. We would suggest 1 as a default choice for the standard deviation of the slopes, which can be implemented with the following:


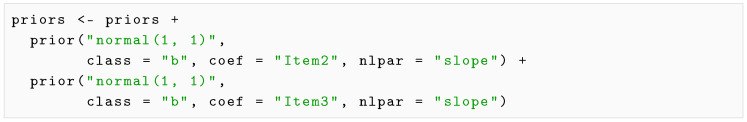


Finally, like with the variance standardization method, the easiness parameter corresponds to the expected count for someone of average ability ($\theta_{i}=0$). Thus, the same distributions may be used to specify weakly informative priors for easiness parameters:


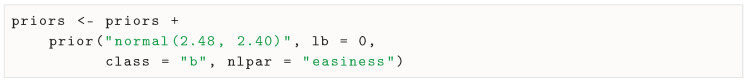


Although this is only one example, these priors led to successful model estimation (per the convergence inspection methods later described) with the marker method in the present dataset.

### 2.5. Model Estimation

We can now estimate the model (with the variance standardization method) using the brm() function:


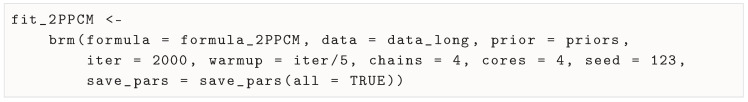


In this function, we specify the formula, the data, the priors, the number of iterations (including warmup), the number of chains, the number of cores, and a seed for reproducibility. We also specify that we want to save all parameters using the save_pars argument (this is useful for certain methods used on brmsfit objects). The model will then be estimated using Hamiltonian Monte Carlo sampling, and the results will be stored in the fit_2PPCM object. The same code can be used to estimate the RPCM, by changing the formula to the formula_RPCM defined earlier. We also estimated it at this stage to later illustrate model comparison possibilities.

## 3. Post-Estimation Analyses

### 3.1. Model Summary

After estimation, a first step that most researchers would take is to inspect the model summary. This can be carried out using the summary() method:


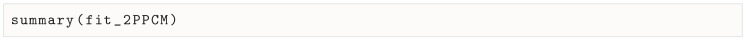


The parameter section of the output is presented below. For users not familiar with Bayesian estimation, it is important to note that, contrary to maximum likelihood estimation, the actual outcome of the estimation for each parameter is a distribution (which is the estimated posterior distribution), not (directly) a point estimate. By default, the summary method provides as a point estimate the mean of the posterior distribution. The estimate error is the standard deviation of the posterior distribution, and the 95% credible intervals are the 2.5% and 97.5% quantiles of the posterior distribution. Finally, some convergence diagnostics are presented for each parameter, which we will discuss in the next section.


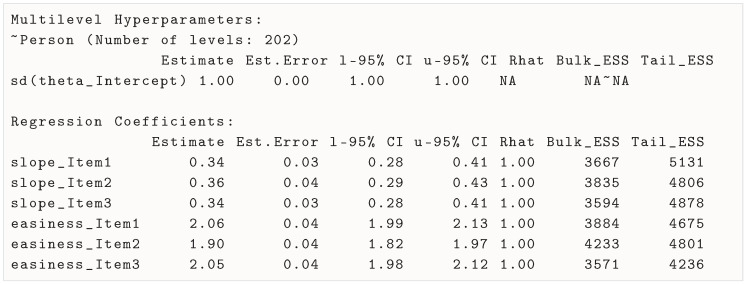


The first part is not informative here, because it presents the value of the standard deviation of the latent variable θ, which was fixed for identification (hence the convergence diagnostics being NA and the estimate error being 0). Note that, if the marker method of identification had been used, the standard deviation of θ would be estimated and presented here.

The second part presents the fixed effects, which are the item parameters. The estimates (as well as their error and credible intervals) are presented on the log scale (not on the scale of the count responses). Easiness parameters are interpreted as the expected log count for someone of average ability ($\theta_{i}=0$). Slope parameters are interpreted as the expected change effect of a one-standard-deviation increase in $\theta_{i}$ on the expected log count. In log-linear models, it is common to exponentiate the estimates when one wants to interpret them. This can be carried out with the following:


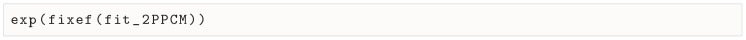


The exponentiated easiness parameters are interpreted as the expected count for someone of average ability ($\theta_{i}=0$), while the exponentiated slope parameters are interpreted as the multiplicative effect of a one-standard-deviation increase in $\theta_{i}$ on the expected count. For example, for item 1 (rope), the expected count of ideas produced by someone of average ability is $e^{2.06}\approx 7.8$, and a one-standard-deviation increase in $\theta_{i}$ multiplies the expected count by $e^{0.34}\approx 1.4$.

### 3.2. Inspecting Model Convergence

In general, brms will output warnings if the model does not converge well. In addition, the summary() method already provides some convergence diagnostics. First, because in MCMC (including HRC) sampling, we generally use multiple estimation chains (in this example, 4), it is important to check that the chains have converged to the same distribution for each parameter. When it is the case, we say that the chains have mixed well. A commonly reported convergence diagnostic is the Gelman–Rubin statistic ($\hat{R}$, Gelman and Rubin 1992), which is a measure of how well the different chains mixed (i.e., how similar the posterior distributions are across chains). It is expected to be close to 1.00 for good convergence across chains. In addition, we also want to make sure that the posterior distribution, which consists, for a parameter, of the values of the parameter for each (post-warmup) iteration of the chain, consists of a large number of independent samples. In other words, we want to make sure that the posterior distribution is not too auto-correlated. This can be assessed by looking at the effective sample size (ESS), which is a measure of the number of independent samples in the posterior distribution. The ESS is expected to be large (e.g., above 400) for good convergence. Bulk ESS values evaluate how well the chains are exploring the bulk of the posterior distribution, while tail ESS values evaluate how well the chains are exploring the tails of the distribution. In general, both ESS values should be above 400 (Vehtari et al. 2021) for all parameters.

Both the $\hat{R}$ and ESS values are presented in the summary output, presented in Section 3.1. In addition, during estimation, the number of divergent transitions (ideally 0) is reported. A divergent transition occurs when the HMC algorithm encounters difficulties navigating the parameter space. This usually happens when the model is poorly specified, the priors are too restrictive, or the parameters are strongly correlated, leading to regions of the posterior distribution that are hard to explore accurately, which can indicate that the model is not well specified or that the priors are too restrictive. In the present examples, we found that the 2PPCM converged well, with $\hat{R}$ values close to 1.00, ESS values well above 400, and no divergent transitions.

Among graphical methods to investigate convergence, it is common to use a trace plot, which shows the value of the parameter at each iteration of the chain, as well as a histogram or density plot of the posterior distribution in each chain. The mcmc_plot() method uses the bayesplot package (Gabry et al. 2019) to easily produce these plots (here, for the item parameters):


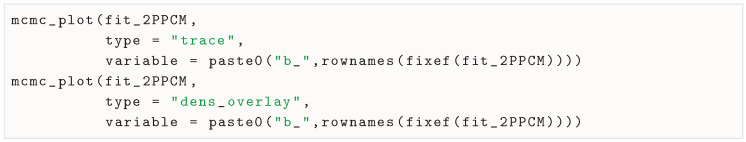


**Figure 1:**
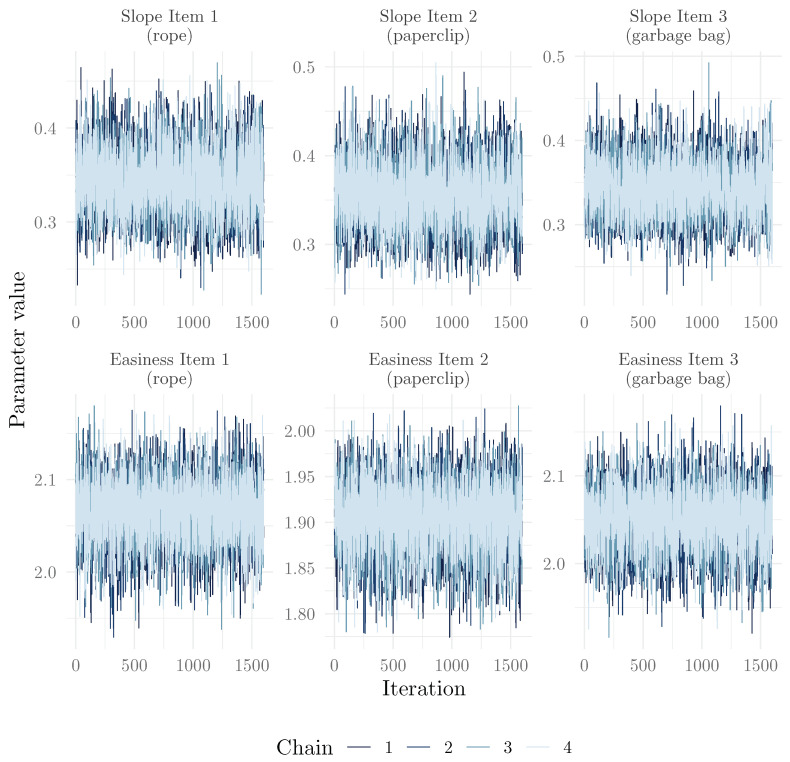
Trace plots for the 6 item parameters of the 2PPCM.

**Figure 2:**
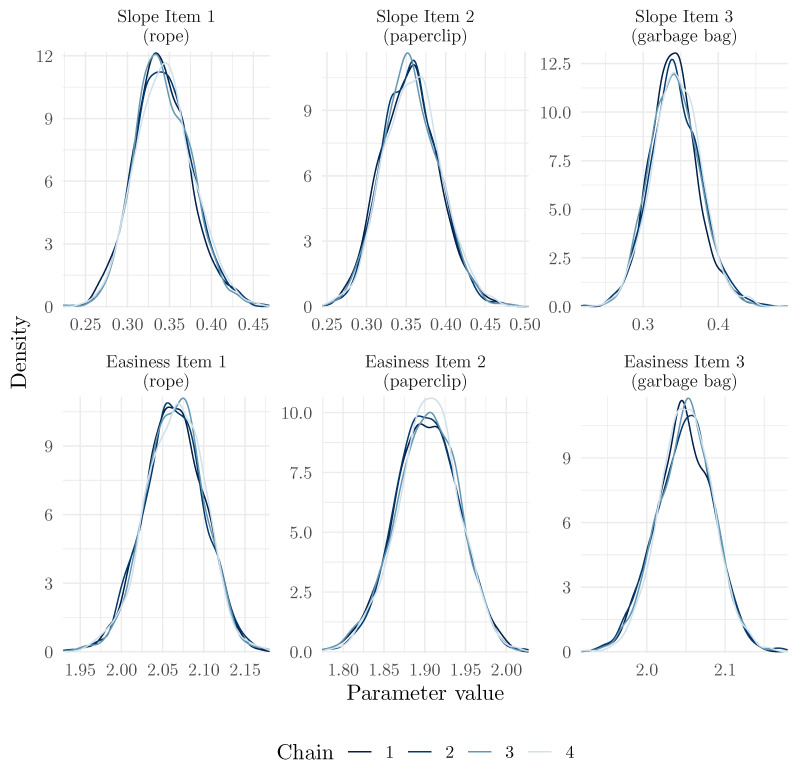
Posterior distributions for the 6 item parameters of the 2PPCM.

We present in Figure 1 and Figure 2, respectively, the trace plots and posterior distributions for the six item parameters. We can see that the chains mixed well, and that the posterior distributions are well behaved. We can also see that the chains explored the tails of the distribution well, as the histograms are well shaped.

In addition to the brms and bayesplot functions and methods, the package shinystan (Gabry and Veen 2022) provides an interactive and convenient way to quickly explore and diagnose potential convergence issues.

If the model does not converge well, it is generally advisable (besides verifying that the model was correctly specified) to try increasing the number of iterations, increasing the number of chains, or using less restrictive priors. For divergent transitions, it is advisable to increase the adapt_delta, which is a tuning parameter for the HMC algorithm that controls how the step size is adapted during sampling. A higher value (e.g., 0.95) makes the HMC sampler more conservative, which increases computation time but can help reduce the number of divergent transitions. This can be carried out by using control = list(adapt_delta = 0.95) in the brm() function.

### 3.3. Extracting Item Parameter Estimates

Item parameter point estimates are already reported when using the summary() method, but they can also be more directly accessed (along with estimate errors) using the fixef() method:


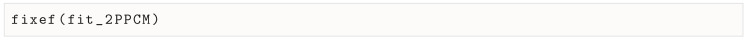


**Figure 3:**
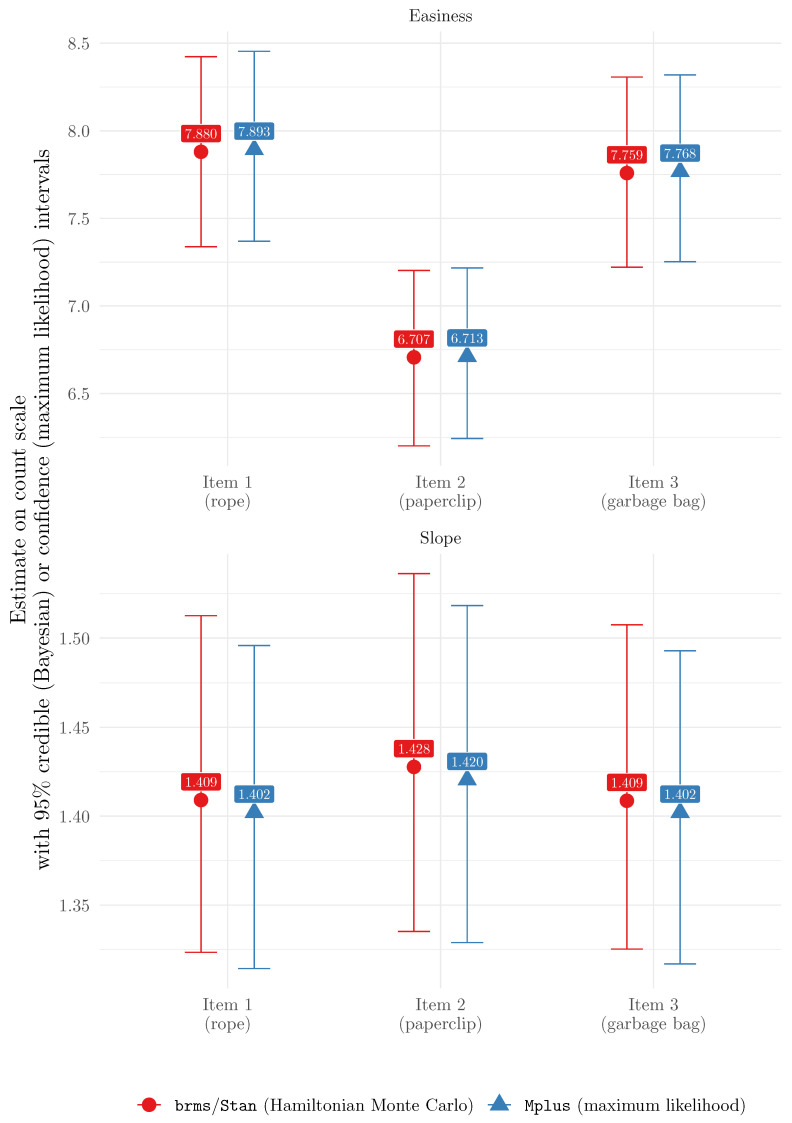
Comparison between item parameter estimates (on original response scale) obtained using Hamiltonian Monte Carlo (brms) and maximum likelihood (Mplus).

As a benchmark, we also estimated the 2PPCM using Mplus (which used maximum likelihood estimation), following the code of the original paper presenting the model (Myszkowski and Storme 2021). The item estimates obtained with brms and Mplus are presented for comparison in Figure 3. We can see that the estimates were very similar, and so were the credible/confidence intervals. This suggests that the 2PPCM can be estimated using brms with results that are very similar to those obtained using maximum likelihood estimation. However, it should be noted that this similarity depends on the priors used in brms. The priors in this example application were purposely weakly informative, and used in order to help model estimation (i.e., they were not used to convey any prior belief about the instrument). More informative priors would lead to different estimates, and thus to potentially less similarity with maximum likelihood methods.

### 3.4. Comparing Models

Model comparisons can be made using a number of methods. Recommended methods include leave-one-out (LOO) cross-validation (Vehtari et al. 2017) and the widely applicable information criterion (WAIC) (Watanabe 2010), both implemented in brms using the loo package (Vehtari et al. 2017). They can be obtained using the following:


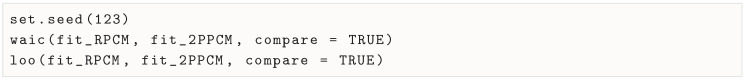


Since both of these methods involve random sampling, it is advisable to set a random seed to obtain reproducible results. Both of these methods provide an estimate of the out-of-sample predictive accuracy of the model through their expected log predictive density (ELPD). A higher ELPD indicates better out-of-sample predictive accuracy. The difference in ELPD between two models is provided when the compare = TRUE argument is used.

In this example dataset, we found that the 2PPCM had a lower ELPD than the RPCM (per both the WAIC and LOOIC), indicating better out-of-sample predictive accuracy for the RPCM. The ELPD difference was −2.0 (SE=0.5) for the WAIC method and −1.9 (SE=0.6) for the LOO method, which suggests that the RPCM is more parsimonious and should be preferred in this dataset. This is not surprising, considering how similar the item slope parameters appeared previously and considering that, contrary to the dataset analyzed in the original paper (Myszkowski and Storme 2021), the dataset at hand contains responses to three tasks of the very same type (all alternate uses), reducing the risk of nuisance factors.

If model evidence (i.e., support for a model in the data) is of more interest than predictive performance (i.e., prediction of out-of-sample data)—this could be the case, if, for example, we were interested in knowing which factor scoring method to use for the examinees in the sample rather than in concluding on which model to use in general with the test—an estimate of the Bayes factor can also be obtained using the bayesfactor() function:


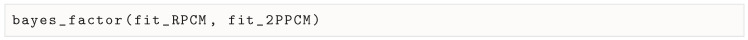


In this dataset, we found that the Bayes factor was 397.95 in favor of the RPCM, which provides substantial evidence for the RPCM being a better-fitting model in this dataset.

Finally, apart from a model comparison approach, and although it is more of a component-wise approach, the hypothesis testing feature of brms can also be used to test whether the slopes differ using a series of pairwise comparisons:


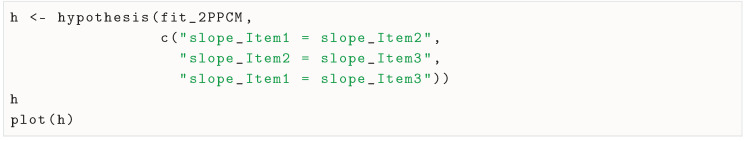


Printing the hypothesis object h will provide the Bayes factor for each comparison, while the plot() method will show the distribution of posterior draws for the slope differences. In this dataset, we found that the 95% credible intervals of slope differences included 0, suggesting no slope differences, which was in line with the RPCM outperforming the 2PPCM in this dataset.

### 3.5. Inspecting Model Fit

#### 3.5.1. Model Fit

**Figure 4:**
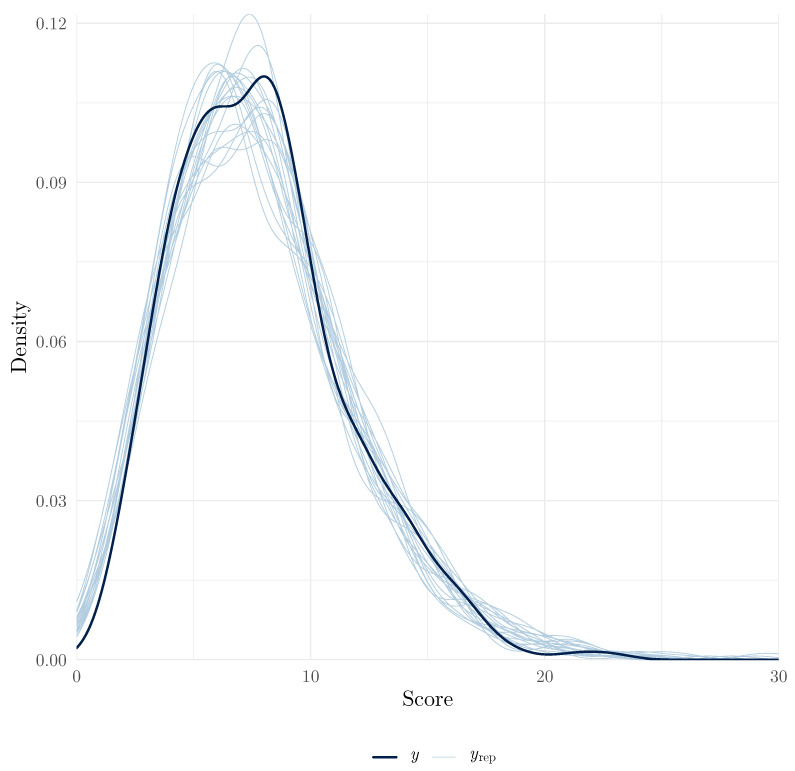
Posterior predictive check for all scores.

Model fit is typically assessed using the posterior predictive checks (PPCs) method, which involves comparing the observed data to data simulated from the posterior predictive distribution. Good model fit is indicated when the observed data are plausible under the model. The pp_check() method uses the bayesplot package to produce a number of plots that can be used to assess this. In the context of IRT, we may want to compare observed score distributions (*y*) and random draws of the posterior predictive distributions ($y_{\mathrm{rep}}$). This plot, presented in Figure 4, can be obtained using the following:


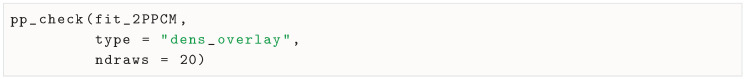


In this plot, we can see that the distribution of observed scores and the posterior predictive distributions are similar, which suggests that the model fits the data well. For a diagnosis of misfit that is less based on graphical examination, it is also possible to generate posterior predictive draws using the posterior_predict() method and to compare these draws to observations numerically. For example, one can verify if the observations are plausible under the model by verifying that they fall within the 95% credible interval of the posterior predictive distribution. We found that only one observation (Item 1, Person 192) fell outside of the 95% credible interval, which suggests good fit (code on the OSF repository).

#### 3.5.2. Item Fit

**Figure 5:**
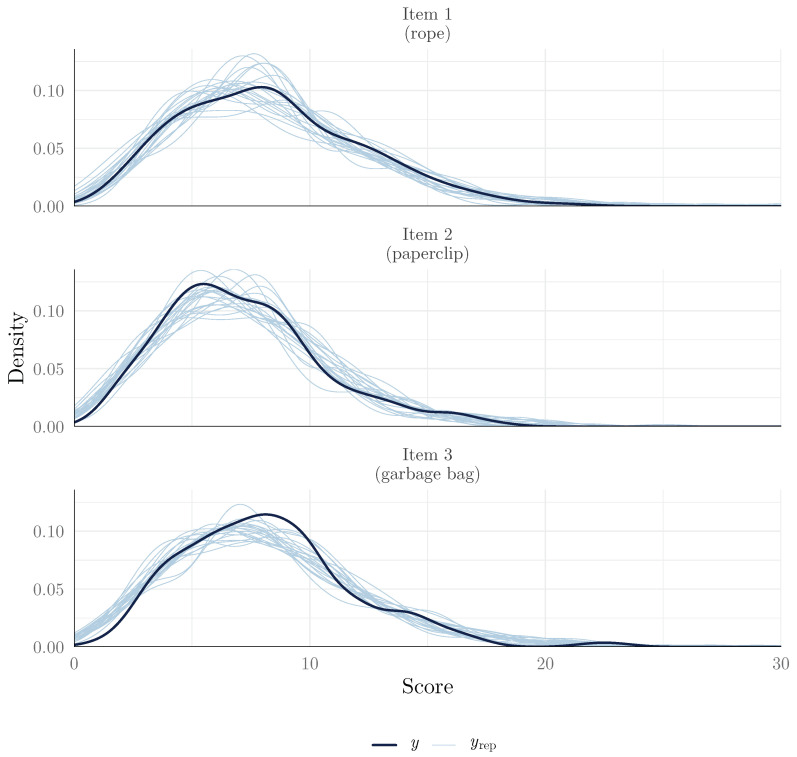
Posterior predictive check (density plot by item).

In the IRT tradition, however, we often focus more on item fit (how well the model fits each item separately). Fortunately, the same kind of approach used for model fit can be used to assess item fit, by specifying the group argument to the Item variable (plot shown in Figure 5):


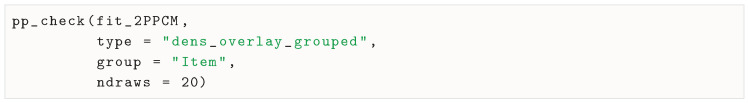


This plot suggests that we found that the observed item scores were plausible under the model, as the observed score distributions were similar to the posterior predictive distributions. This suggests that the 2PPCM is a good fit for the data.

#### 3.5.3. Person Fit

It is also possible to use the same kind of procedure to inspect person fit. This can be carried out by specifying the group argument to the Person variable. Because of the number of cases, however, it can be useful to use the newdata argument to specify a subset of the data to plot. For example, to plot the posterior predictive distributions for the first five participants, we can use the following:


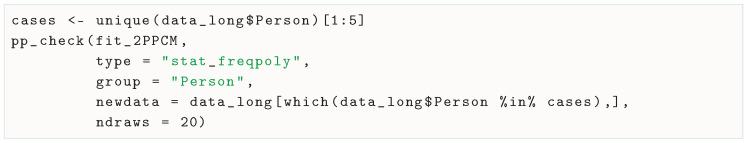


**Figure 6:**
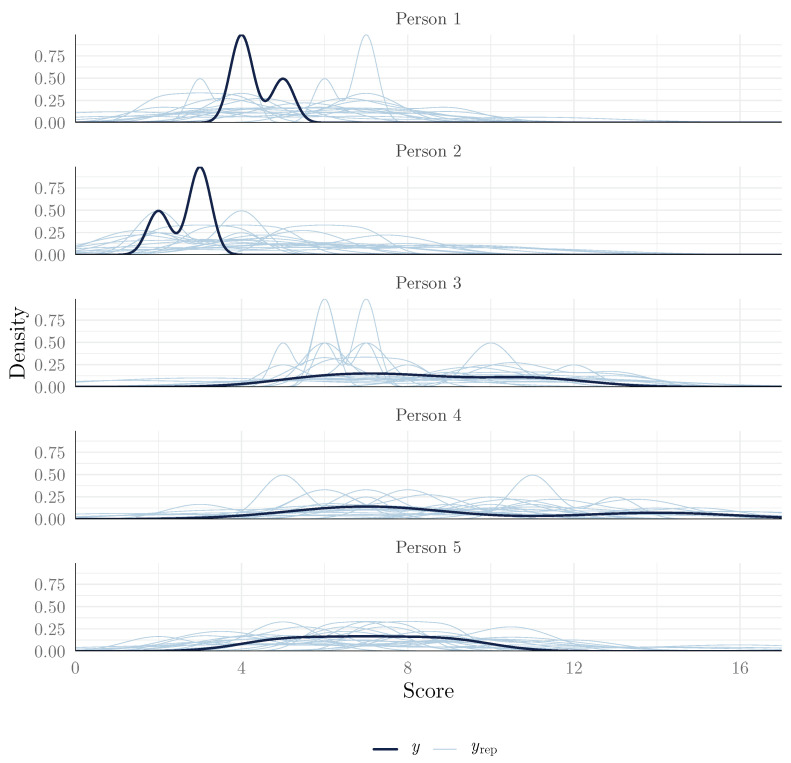
Posterior predictive check (density plot by person).

We show in Figure 6 the posterior predictive distributions for the first five participants as an example. This plot suggests that their observed scores were plausible under the model. To note, in this dataset, there are only three scores per person, so the observed scores are subject to greater sampling variability than when using posterior predictive checks by item in the previous section. Thus, more discrepancies between the observed and posterior predictive distributions are to be expected than before.

#### 3.5.4. Covariate-Adjusted Frequency Plots

**Figure 7:**
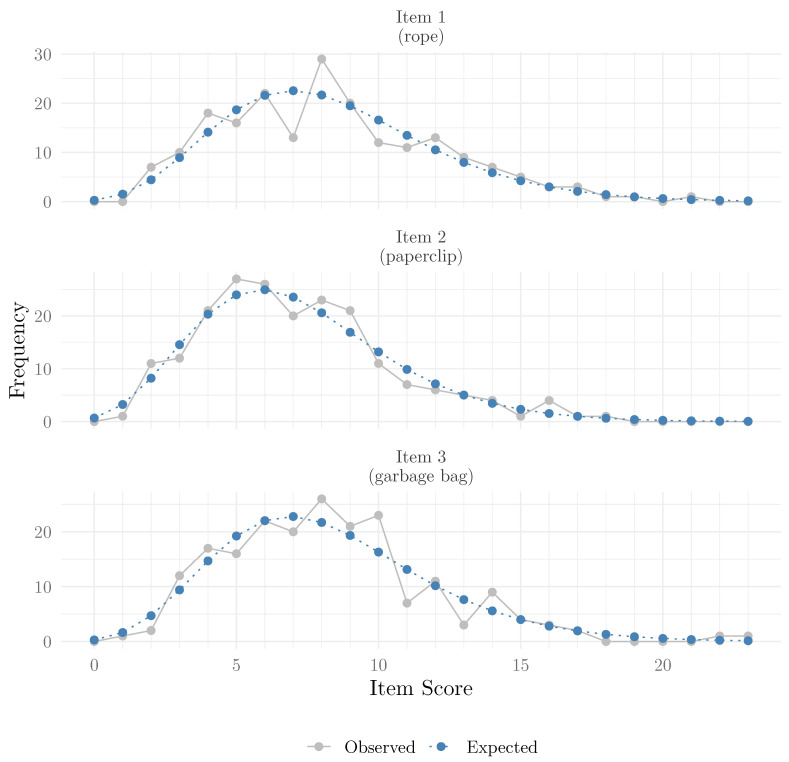
Covariate-adjusted frequency plots by item.

Another option to inspect or illustrate model fit that has been used in the context of IRT for count responses (Forthmann et al. 2019, 2018; Jendryczko et al. 2020) is the covariate-adjusted frequency plot (Holling et al. 2016). This plot compares the observed frequencies to the expected frequencies for each score. While the observed frequencies are directly observed in the data, the expected frequencies are obtained by first predicting the expected means, which correspond to (predicted) rate parameters of the Poisson distribution. This can be carried out using the fitted() method). Afterwards, the expected frequencies are calculated by summing, for a given possible score, the probability mass of the Poisson distribution across all the expected means, which can be performed using the dpois() function. Below, we show how to obtain observed and expected frequencies for Item 1. In Figure 7, we show the covariate-adjusted frequency plot for all items (full code on OSF).


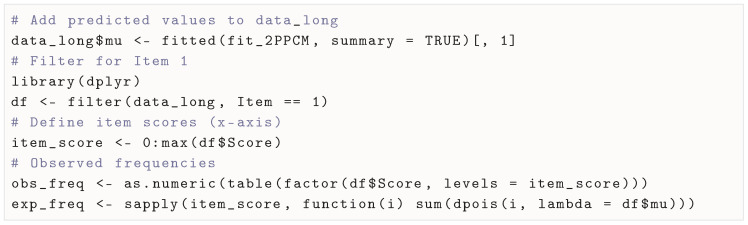


If the model fits the data well, the expected and observed frequencies should be close. In this case, we can see that the 2PPCM seems to fit the data well.

### 3.6. Inspecting Item Response Functions

Once item parameter estimates are extracted, we can use the estimates for a given item to calculate (and plot) the item response function in a range of plausible theta values (e.g., −3 to 3) by applying the response model formula in Equation (4). For example, for item 1, we can use the following:


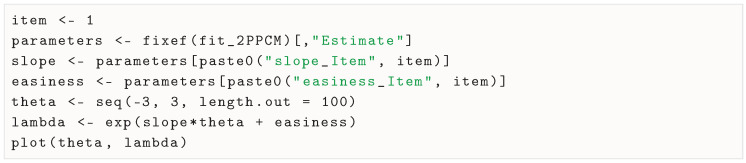


**Figure 8:**
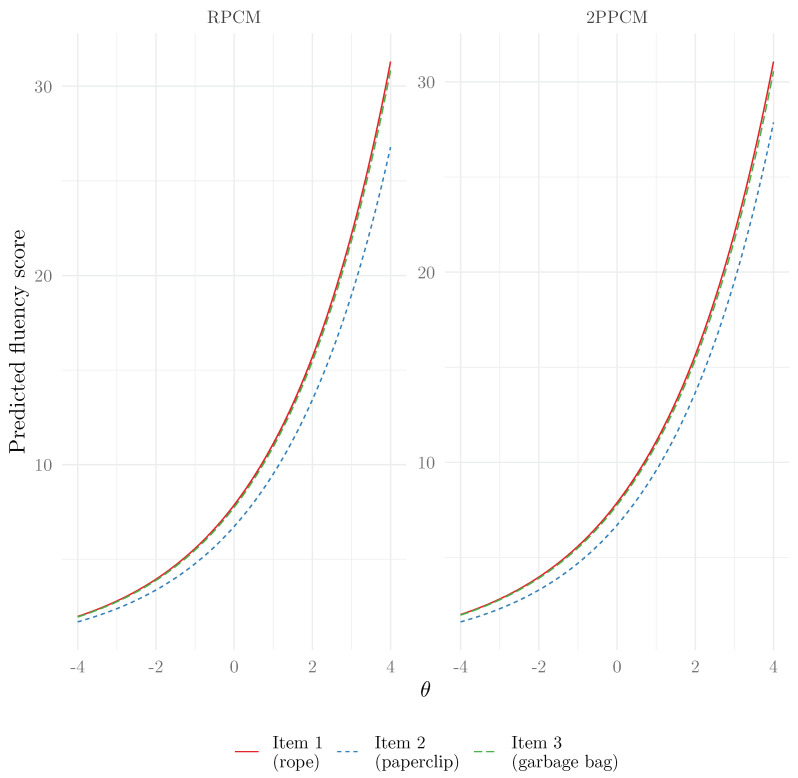
Item response functions of the RPCM and 2PPCM.

We show in Figure 8 the item response functions for all items and for both the RPCM and the 2PPCM. In line with what was seen in the parameter estimates of the 2PPCM, the item response functions have slopes that are very close, but item 2 (paperclip) seems more difficult than the other items. We may speculate that this higher difficulty is attributable to the object itself being less versatile (i.e., people tend to use paperclips for a smaller number of uses than the other objects).

To note, although it can hardly be seen in this case due to the similarity among the discrimination parameters, the item response functions of the 2PPCM are intersecting (i.e., the items do not present invariant item ordering), contrary to those of the RPCM (Myszkowski 2024).

### 3.7. Calculating Test and Item Information

In IRT, information $I(\theta_{i})$ is used to quantify the amount of information that a test (or an item) provides about a person’s ability $\theta_{i}$. Since information functions have not been presented for the 2PPCM in the literature, we provide the formula for the 2PPCM here. Fisher information about a parameter (here $\theta_{i}$) is defined as the negative of the expectation of the second derivative of the log-likehood with respect to it:

(6) $$I\left(\theta_{i}\right)=-E\left[\frac{\partial^{2}\log L\left(\lambda_{ij}\right)}{\partial \theta_{i}^{2}}\right]=-E\left[\frac{\partial^{2}\ell \left(\lambda_{ij}\right)}{\partial \theta_{i}^{2}}\right]$$

By calculation of the first and second derivative (further discussed in Appendix A), we obtain that the test information function $I(\theta_{i})$ is as follows:

(7) $$I\left(\theta_{i}\right)=\sum_{j=1}^{m}a_{j}^{2}\lambda_{ij}=\sum_{j=1}^{m}a_{j}^{2}e^{b_{j}+a_{j}\theta_{i}},$$

and the item information function $I_{j}\left(\theta_{i}\right)$ is as follows:

(8) $$I_{j}\left(\theta_{i}\right)=a_{j}^{2}\lambda_{ij}=a_{j}^{2}e^{b_{j}+a_{j}\theta_{i}}.$$

To note, for the RPCM, these formulae are the same, but the discrimination parameters do not vary by item. We can note that information increases with the (squared) discrimination parameter $a_{j}$, with the easiness parameter $b_{j}$, and with the ability parameter $\theta_{i}$. In other words, all else being equal, items that are easy and have high discrimination parameters provide more information, and more information is provided about persons with high ability parameters (this last point is in contrast with logistic models, where information is maximal when a person’s ability is close to the item’s difficulty).

Because there is no optimum of information, one might argue that examining the information function is less useful than in logistic models. However, its calculation could be important in contexts such as adaptive testing or optimal test design, where items may be selected based on the expected information that they provide about a person’s location. Furthermore, whereas, in the RPCM, items with higher easiness parameters provide more information at any given $\theta_{i}$ (which makes item selection based on information straightforward, as it implies that items with higher easiness should be preferred), this is not necessarily the case in the 2PPCM, due to the introduction of variable discrimination parameters (e.g., an item may be very easy but provide low information if its discrimination parameter is low).

To compute test information functions, we can use point estimates of the parameters (which we previously extracted). It is typical to examine item information through item information curves (IICs), which show how much information an item provides for a range of plausible θ values. We can calculate the IIC for item 1 (reusing the previously extracted parameters) using Equation (8), and plot it using the following code:


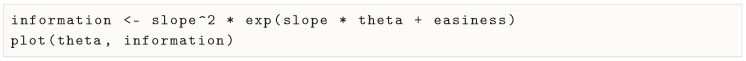


**Figure 9:**
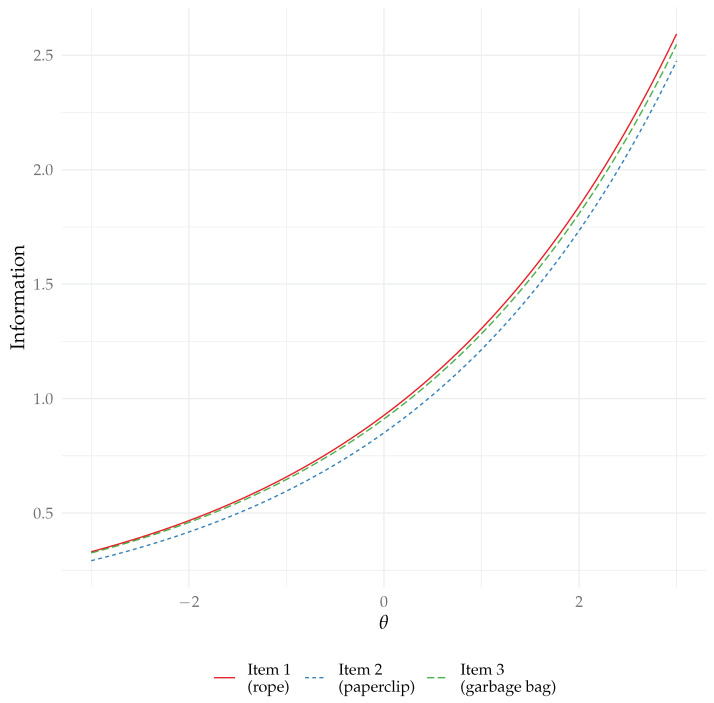
Item information curves.

We show in Figure 9 the item information curve for all items.

The total test information can simply be obtained by summing item information:


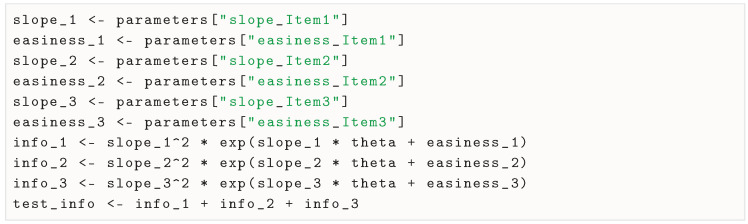


The calculation of test information can also be useful in the calculation of expected (i.e., marginal) reliability.

### 3.8. Examining Equidispersion

The Poisson distribution assumes that the variance is equal to the mean, an assumption known as equidispersion. Violations of this assumption lead to biased estimates of reliability and standard errors, with estimates being more conservative in the case of underdispersion, and more liberal in the case of overdispersion (Forthmann et al. 2019). Hence, underdispersion tends to be considered a less important problem than overdispersion (Jendryczko et al. 2020).

As suggested for the RPCM (Baghaei and Doebler 2019) and used for the 2PPCM (Myszkowski and Storme 2021), this assumption can be checked using the dispersion parameter ϕ. The overall dispersion parameter can be calculated using the following formula:

(9) $$\phi =\frac{1}{n\cdot m}\sum_{i=1}^{n}\sum_{j=1}^{m}\frac{{\left(y_{ij}-{\hat{y}}_{ij}\right)}^{2}}{{\hat{y}}_{ij}}.$$

Equidispersion implies that ϕ=1, underdispersion implies that ϕ<1, and overdispersion implies that ϕ>1. To compute it from our brmsfit object, we retrieve predictions in the sample using the fitted() method (which provides the posterior mean for each observation):


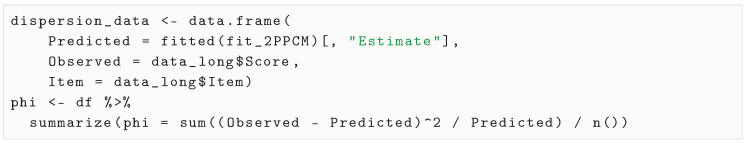


We found that the dispersion parameter was 0.469, which indicates underdispersion. In a similar manner, we may want to calculate dispersion by item:    

(10) $$\phi_{j}=\frac{1}{n}\sum_{i=1}^{n}\frac{{\left(y_{ij}-{\hat{y}}_{ij}\right)}^{2}}{{\hat{y}}_{ij}}.$$

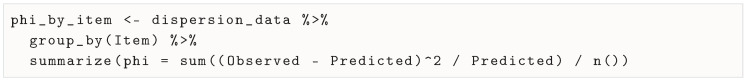


All items showed underdispersion ($\phi_{1}=0.565$, $\phi_{2}=0.438$, $\phi_{3}=0.405$). Other packages like DHARMa (Hartig 2024) use different formulae to test for non-equidispersion and can also be used on brms models’ objects:


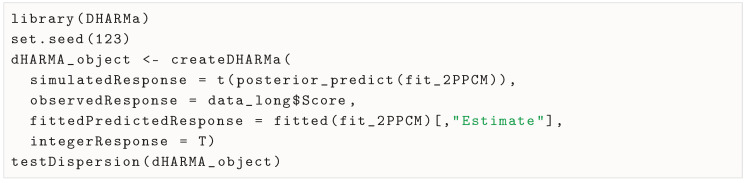


The DHARMa dispersion test indicates significant underdispersion ($\phi^{\prime }=0.380$, p<.001), which is in line with the previous calculation. When looking at dispersion by item (code provided in OSF repository), all items show underdispersion ($\phi_{1}^{\prime }=0.452$, p<.001, $\phi_{2}^{\prime }\;=\;0.356,p<.001$, $\phi_{3}^{\prime }=0.331,p<.001$).

As was proposed for the 2PPCM and RPCM (Myszkowski and Storme 2021), for Poisson models, a graphical representation of the dispersion by item can be obtained using Pearson residuals ($r=\frac{y_{ij}-{\hat{y}}_{ij}}{\sqrt{{\hat{y}}_{ij}}}$) as a function of the predicted values. Ideally, the residuals should be evenly distributed around 0, with no clear trend or structure. When this is not the case, this plot can allow for detection of systematic patterns in dispersion (e.g., predicted score values where the model consistently overpredicts or underpredicts). Pearson residuals are obtained using the following:


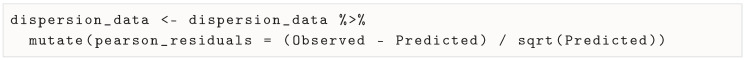


**Figure 10:**
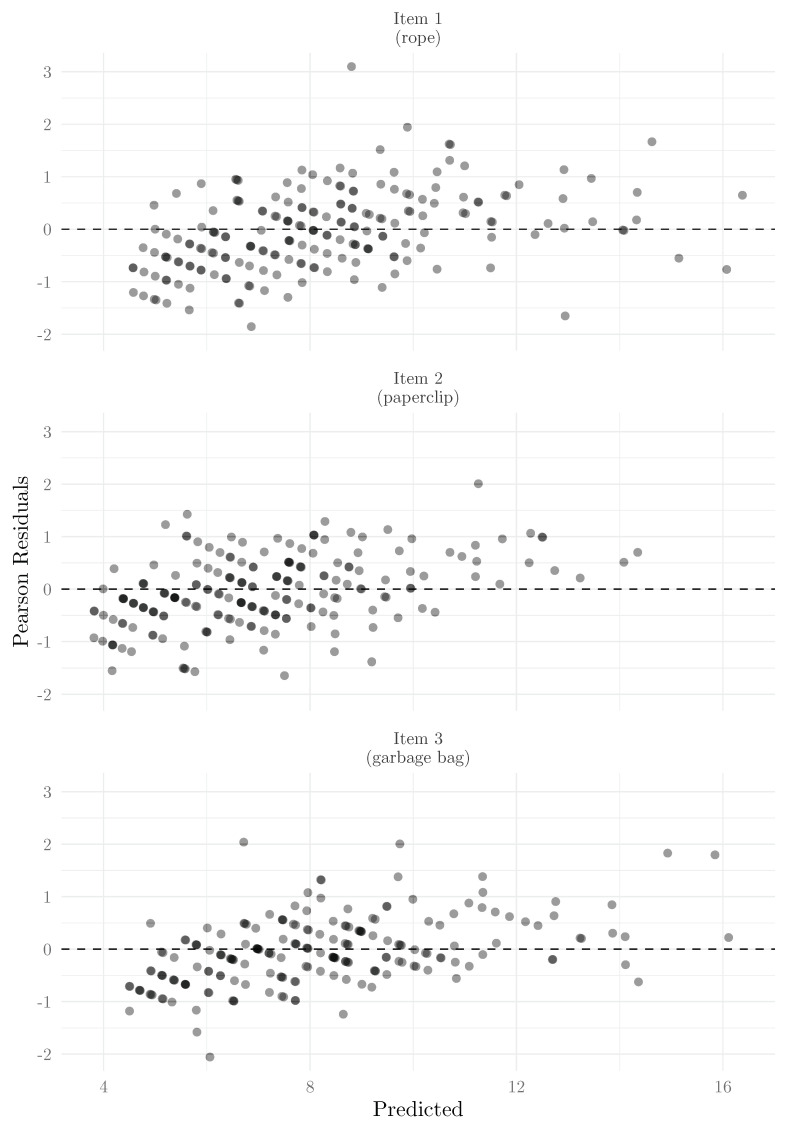
Pearson residuals as a function of the predicted values.

We show in Figure 10 the Pearson residuals as a function of the predicted values by item.

Although the residuals appear relatively stable and clustered around 0, we can note, overall, an increasing trend, which indicates that the model tends to systematically overpredict for low predicted scores and underpredict for high predicted scores.

### 3.9. Extracting Factor Scores

Point estimates, standard errors and credible intervals for $\theta_{i}$ can be obtained (notably) using the ranef() method:


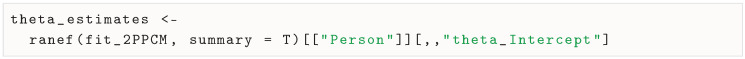


To fully leverage the Bayesian framework when describing a person’s level, one may be interested in extracting the posterior distribution of $\theta_{i}$ using posterior draws. This can be performed using the as_draws set of functions. Here, we show how to extract the posterior distribution of θ across all participants, chains and iterations (without the warmup):


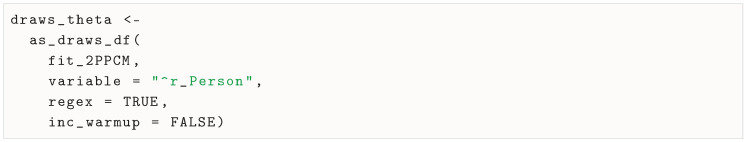


**Figure 11:**
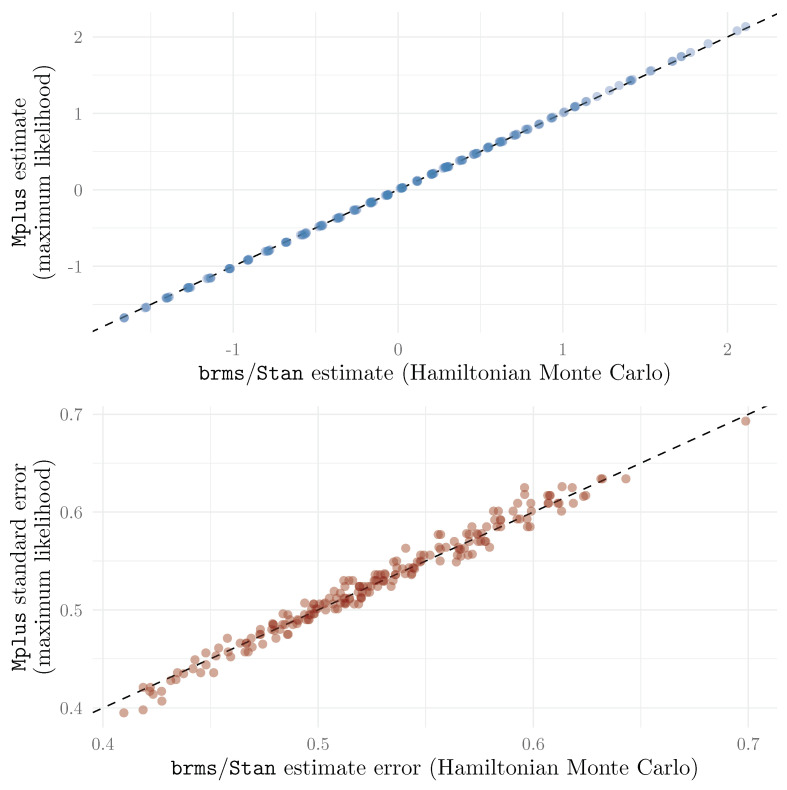
Comparison of θ estimates and estimate errors (with identity function for reference).

We show a comparison of the $\theta_{i}$ estimates obtained through ML estimation in Mplus and brms in Figure 11. We can see that the estimates are practically identical (r>0.999), and that the errors (standard errors for Mplus, and standard deviation of the posterior distribution for brms) are also very similar r=0.989).

### 3.10. Calculating Reliability

There are several approaches to the calculation of reliability in the context of IRT models. One common approach, which is the one used in the empirical_rxx() function of the mirt package (Chalmers 2012), is based on the typical formula for reliability in classical test theory, which is the ratio of the true score variance to the sum of the true score variance and the error variance:

(11) $$\mathrm{Empirical}\;\mathrm{reliability}=\frac{\mathrm{VAR}(\hat{\theta })}{\mathrm{VAR}\left(\hat{\theta }\right)+\mathrm{VAR}\left(\hat{\mathrm{Error}}\right)}.$$

In the context of Bayesian IRT, for group-level empirical reliability, we can use as a proxy for the true score variance the variance of the $\theta_{i}$ estimates (which implies that this method is dependent upon the method used to obtain a point estimate from the posterior distribution), and as a proxy for the error variance the mean of the square of the error estimates:


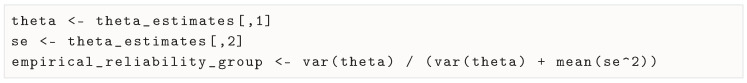


In this dataset, we found that the group-level empirical reliability was 0.716. For person-level reliability, we can use the same method, but using the squared error for the person instead of the mean squared error:


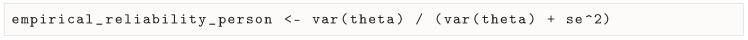


**Figure 12:**
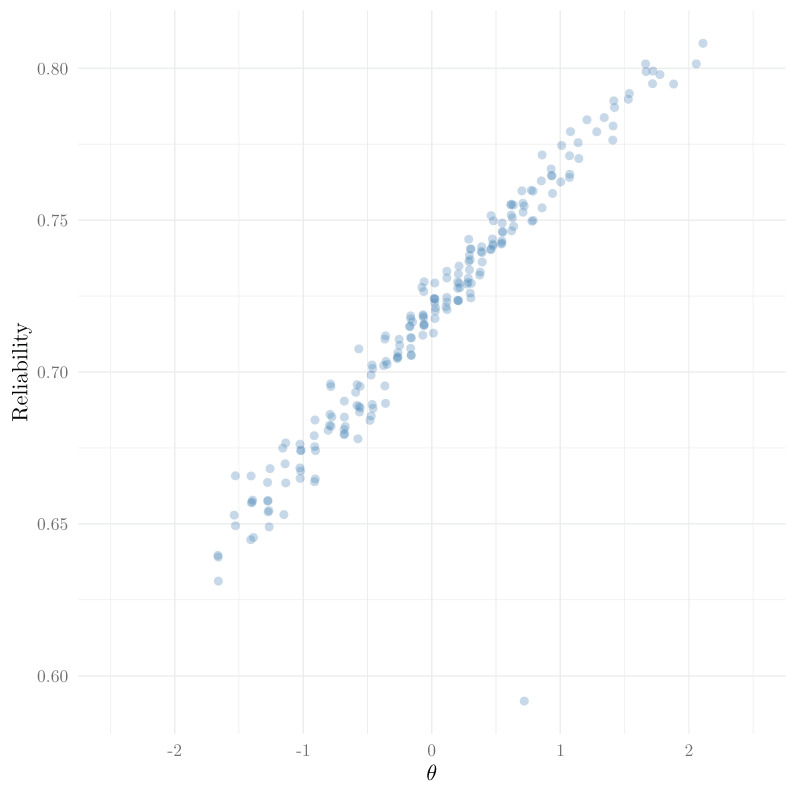
Reliability as a function of θ.

We show a scatter plot of reliability as a function of θ in Figure 12. In line with item information curves, we can see that empirical reliability tends to increase with θ in the 2PPCM. Expectedly, the examinee that had only responded to one item has a lower reliability that they would have with scores for all items, and is therefore an outlier in this plot.

### 3.11. Suggested Workflow

To wrap up this section, we present a suggested workflow for the estimation of Poisson IRT models using brms. After the package has been installed and tested (e.g., using an example in the package documentation), we would recommend specifying the 2PPCM using the code we provided using the default priors. After estimation, one should carefully inspect convergence (e.g., divergent transitions, low ESS, poor mixing of the chains), and in case convergence issues are found, focus on using informative priors (especially for the discrimination parameters). If the model still does not converge, then try increasing the number of iterations, the number of chains, and/or the adapt_delta parameter.

Once a model has been estimated and the output and summary show no (or nearly no) convergence issues, we would suggest estimating other candidate models (e.g., the RPCM). Once all candidate models have been estimated, compare their fit and select the model with the best fit. Use the covariate-adjusted frequency plots and posterior predictive checks to inspect the fit of the selected model. Calculate dispersion and use the Pearson residuals to inspect the equidispersion assumption.

Afterwards, if the model is to be used for scoring persons, extract the factor scores and their errors (or entire posterior distributions). If the model is used to investigate the properties of the test, to refine the test, discard items or assemble new tests, then primarily inspect the item response functions and item information curves, and calculate the overall test reliability.

## 4. Discussion

### 4.1. Summary

In this paper, we used brms to estimate Poisson log-linear IRT models, including the RPCM (Rasch 1960) and the 2PPCM (Myszkowski and Storme 2021). Mplus is based on a generalized structural equation modeling framework, while brms is based on a generalized multilevel modeling framework, which makes the two packages different in terms of syntax and structure. In spite of this, model specification is comparably easy (if not easier) in brms, provided there is some familiarity with R and the Bayesian framework. In the dataset that we analyzed, it was necessary to use weakly informative priors for discrimination parameters in lieu of the default flat priors of brms in order to ensure model convergence, but this may due to the properties of the dataset. Even though they may not be necessary in all cases, we proposed weakly informative priors for item parameters, which were based on the expected range of the parameters. They allowed the 2PPCM to converge successfully, as evidenced by our convergence checks.

We showed that the estimates obtained using brms were very similar to those obtained using Mplus, which suggests that brms can be used to estimate Poisson IRT models with results that are very similar to those obtained using maximum likelihood estimation in other cases. The similarity between the estimates found through HMC and maximum likelihood estimation can be seen as a cross-validation of the results, but, in and of itself, it does not necessarily encourage the use of one method over the other. It is thus important to note that the Bayesian framework offers a number of advantages over maximum likelihood estimation that we did not explore, including the ability to incorporate prior information (e.g., if pre-test evidence suggests that a given item is likely to be more difficult than another, this can be incorporated in the model), the ability to quantify uncertainty using a full posterior distribution (rather than point estimates and standard errors, which is particularly important for posterior distributions that are skewed or multimodal), or the ability to safeguard against over-fitting or extreme estimates (which is particularly relevant with small sample sizes) through the use of regularization (Bayesian estimation naturally shrinks parameters towards their prior). In other words, the implementation showed here does not explore the full potential of the Bayesian framework as it is implemented in brms and Stan and shall be considered a starting point.

After estimation, we showed how to obtain the typical psychometric indices that are generally calculated in IRT analyses, including item response functions, information functions and reliability. We showed how to diagnose issues of non-equidispersion, which are common in Poisson models. In addition, we showed that it was possible, within the Bayesian framework, to study model fit through posterior predictive checks—which can be implemented to check fit at the model level, at the item level, and at the person level—and to compare the fit of different models. We also showed how to extract factor scores and measurement error estimates, and found that the estimates obtained using brms—for both the factor scores and their errors—were very close to those obtained using maximum likelihood estimation in Mplus. Overall, we believe that brms is a very promising tool for the estimation of Poisson IRT models and its various extensions.

In this study, in contrast with the results obtained in the original paper presenting the 2PPCM (Myszkowski and Storme 2021), the RPCM had better fit. Although we previously noted that, in divergent thinking tests, it is plausible to have items with different discrimination (for example, in the case of alternate uses tasks, because nuisance factors like social inhibition and domain expertise could partially explain scores), where a 2-parameter model would be called for, it appears that this was not the case with these three items (rope, paperclip and garbage bag), making the 2PPCM a less parsimonious choice than the RPCM. We might speculate that the 2PPCM was not a better fit in this case because the items all used the same prompt (finding alternate uses) with objects that were all relatively similar in their banality (i.e., no object would tap into a specific domain of expertise). Although this is likely specific to each test, future research on divergent thinking tasks could help determine whether, in general, one-parameter models like the RPCM are sufficient and in which situations they are not. We also found violations of equidispersion in these data, which indicates that the Poisson distribution may not be a good choice for it. As we discuss later, models that accommodate underdispersion, such as models that use the Conway–Maxwell–Poisson distribution (Beisemann 2022; Forthmann et al. 2019) may be more appropriate than Poisson models in this case. Finally, we found that the test had satisfactory reliability overall, especially for a test that consisted of three items.

### 4.2. Limitations and Future Directions

There are still some limitations to the current implementation. First, there may be some specificities to the current dataset that may not generalize to other datasets: It is possible that the dataset used in this paper was too small to trigger estimation issues that may arise in larger datasets. It is also possible that there are situations (e.g., missing data designs, outliers) where estimating models with brms may be more challenging, or may necessitate more specific priors.

Second, although we attempted to highlight the user-friendly aspects of brms, the current implementation still requires some familiarity with R, especially if one wants to customize outputs (e.g., generate layered plots). In comparison, packages focused on IRT like mirt (Chalmers 2012), flirt (Jeon and Rijmen 2016) or ltm (Rizopoulos 2006) would be certainly considered more user-friendly. In this context, we consider that brms is a user-friendly and flexible tool for their implementation.

Third, a limitation of this paper is that it is only a starting point, because there are a number of extensions to count IRT analysis that we did not develop and present here. Indeed, the 2PPCM is only one of several extensions that have been recently proposed for count responses. For example, it has been proposed that distributions that accommodate non-equidispersion be used, such as the negative binomial distribution (Hung 2012; Mutz and Daniel 2018) or the Conway–Maxwell–Poisson distribution (Forthmann et al. 2019, 2024). Notably, the 2-parameter Conway–Maxwell–Poisson model (2PCMPM; Beisemann 2022) uses the same response function as the 2PPCM, but assumes that scores follow a Conway–Maxwell–Poisson distribution, which accommodates both under- and overdispersion. Importantly, the dispersion parameter of the distribution is item-specific, allowing items to be under- or overdispersed. The Conway–Maxwell–Poisson distribution having the Poisson distribution as a special case, the 2PCMPM is a further generalization of the 2PPCM. Future research may explore how to estimate the 2PCMPM using brms and Stan, and how it produces estimates that are consistent with maximum likelihood estimation (see Beisemann 2022). In addition, it has been proposed that logistic (rather than exponential) inverse link functions be used (Doebler et al. 2014), in order to account for the fact that, even at very high ability levels, expected counts may not grow indefinitely (e.g., in divergent thinking tasks, even someone with very high idea generation abilities is limited by their writing speed). Even though they were not covered in this paper, we believe that these extensions, in both distributional assumptions and response function, could be implemented in brms the future.

Fourth, related to the previous point, there are other assumptions, more common to measurement models, that we did not cover in this paper. For example, it is assumed that persons with the same ability level have the same expected score, regardless of their other characteristics (e.g., demographic category), an assumption known as measurement invariance, which in IRT is generally investigated at the item level through differential item functioning (DIF) analysis. In this paper, we did not cover how to use brms to test for DIF in count IRT models. However, we expect that it should be feasible using person-by-item covariates (Boeck et al. 2011; Bürkner 2021). Also, we did not cover how to use brms to test for local independence, which is another important assumption in IRT. At this stage, we believe that this could be carried out (at least in a rudimentary way) by examining correlations between the distributions of residuals per item. It may also be possible in brms to add specific factors and create a bifactor/testlet structure (see Myszkowski and Storme 2021, for a bifactor 2PPCM), which would allow for accommodating local dependencies.

Fifth, there are also a number of extensions of the 2PPCM itself that could be implemented in the future. These notably include explanatory IRT models (De Boeck and Wilson 2004), see Forthmann et al. (2024) for an application on count data. brms also accommodates multivariate models, which indicates, for example, that compensatory models, which have been discussed as useful extensions to the 2PPCM (notably bifactor models, see Myszkowski and Storme 2021), could probably be estimated with it in the future. In addition, models with mixed formats (e.g., models that integrate both count and non-count variables) may also be possible and could be very useful in creativity research, where different kinds of tests are used with different forms of scoring (e.g., originality scoring and fluency scoring). Finally, models that allow for incorporating sequential dependencies that come from creative sprees (i.e., a person who has just produced many ideas on an item may be prone to produce a lot to the next item, over and beyond their latent fluency) or depletion (i.e., a person who has just produced many ideas on an item may be prone to produce less to the next item, over and beyond their latent fluency) could be studied using an auto-regressive IRT framework (Myszkowski and Storme 2024; Tang et al. 2020).

Sixth, although the Bayesian tradition often relies on graphical methods for model fit, we may suggest focusing on developing additional methods to investigate model/item/person fit that produces numeric indices that can be compared to cut-offs and benchmarks. This can notably be useful when the number of cases and/or items is large, or when one wants to implement a more automated or objective decision (e.g., when filtering out items in a test development stage). For example, we would suggest defining a discrepancy function in order to obtain a posterior predictive *p*-value (Gelman et al. 1996)—see Fox (2010) for a discussion of these methods in IRT contexts, which could be used to further discuss item fit. As a discrepancy function, we would recommend the 5-binned $\chi^{2}$ statistic proposed in the frequentist tradition for the RPCM by Baghaei and Doebler (2019). This would allow for obtaining a numeric index of item fit that could be used to filter out items that do not fit the model.

Finally, we did not investigate how to fully leverage the Bayesian framework and its specificities in this paper. For example, we did not investigate how priors could be used for regularization (e.g., to make the estimation process more robust to over-fitting, which appeared to be a risk for the 2PPCM in the current dataset). One could imagine a re-specification of the 2PPCM to include a common discrimination parameter (e.g., mean discrimination) and item-specific discrimination parameter deviations. Narrow priors around 0 could then be used to regularize these discrimination deviations towards 0. Alternatively, approaches like spike-and-slab priors (Ishwaran and Rao 2005)—which have been used for variable selection in IRT contexts (e.g., Jeon et al. 2021)—could also be used to trap discrimination deviation parameters when they are close to 0, so as to discard them and simplify the response model on an item-specific basis. These applications could also be further developed for the detection of DIF (and therefore, item/test bias) in count IRT models.

## 5. Conclusions

We have shown that the brms package and Stan can be used to fit Poisson IRT models and to perform the wide range of psychometric analyses generally performed in and expected from a typical IRT analysis. Overall, we found that brms greatly facilitates the implementation of Bayesian IRT models and the calculation of various IRT indices without having to learn Stan, although some understanding of the Bayesian framework, IRT and R is certainly recommended.

In this example dataset, we found that the 2PPCM did not fit the data better than the RPCM, which indicates that the items had similar discrimination parameters. We also found that the test had satisfactory reliability, especially considering that it is a fluency test with only three items, and that the estimate of reliability obtained was conservative due to underdispersion. Because these results may not reflect the results of other fluency tests, where 2-parameter count models can outperform Rasch models (e.g., Myszkowski and Storme 2021), and because the assumption of equal discriminations is worth verifying even when it is not violated, we hope that this paper will incite researchers to consider both a Bayesian framework and 2-parameter count IRT models when they analyze divergent thinking fluency tests, and fluency tests in general.

## Figures and Tables

**Table 1:** Summary of the arguments used in the bf() function.

Argument	Description	Use
Score ~ 0 + easiness + slope * theta	Outcome formula	Defines the outcome variable (Score) as a function of item and person parameters (easiness, slope, and theta). The intercept is omitted with 0 +.
theta ~ 0 + (1 | Person)	Latent variable specification	Defines theta as a random intercept for each Person, with the population mean of theta set to 0 with 0 +.
easiness ~ 0 + Item	Fixed effects for easiness	The easiness parameter varies by Item.
slope ~ 0 + Item	Fixed effects for slope	The slope parameter varies by Item.
nl = TRUE	Nonlinear model indicator	Allows for flexible specification of relationships between parameters.
family = poisson(link = "log")	Outcome variable distribution and link	Specifies that the outcome variable follows a Poisson distribution with a log link function.

## Data Availability

The data used in this paper are available at https://osf.io/a9qnc (accessed on 15 February 2025).

## Appendix A. Information Function of the 2PPCM and RPCM

From Equation (6), item information is the negative of the expectation of the second derivative of the log-likehood with respect to the latent ability $\theta_{i}$. To calculate it, we first need to find the likelihood, then the log-likelihood. Afterwards, we will calculate the first derivative of the log-likelihood, and then the second derivative. Finally, we will calculate the negative of its expectation. More details of these calculations are available on OSF.

### Appendix A.1. Likelihood

From the probability mass function of the Poisson distribution, we obtain the likelihood:

(A1) $$L\left(\lambda_{ij}\right)=\prod_{i=1}^{n}\prod_{j=1}^{m}\frac{\lambda_{ij}^{x_{ij}}e^{-\lambda_{ij}}}{x_{ij}!}.$$

### Appendix A.2. Log-Likelihood

From the likelihood, we obtain the log-likelihood:

(A2) $$\begin{array}{cc} \ell (\lambda_{ij}) & =\log\left(\prod_{i=1}^{n}\prod_{j=1}^{m}\frac{\lambda_{ij}^{x_{ij}}e^{-\lambda_{ij}}}{x_{ij}!}\right) \end{array}$$

(A3) $$\begin{array}{cc} \; & =\sum_{i=1}^{n}\sum_{j=1}^{m}\left[x_{ij}\log\left(\lambda_{ij}\right)-\lambda_{ij}-\log\left(x_{ij}!\right)\right]. \end{array}$$

### Appendix A.3. First Derivative of the Log-Likelihood

We then derive the log-likelihood with respect to $\lambda_{ij}$, using the response model formulation in Equation (4):

(A4) $$\begin{array}{cc} \frac{\partial \ell (\lambda_{ij})}{\partial \theta_{i}} & =\frac{\partial }{\partial \theta_{i}}\sum_{j=1}^{m}\left[x_{ij}\log\left(\lambda_{ij}\right)-\lambda_{ij}-\log(x_{ij}!\right] \end{array}$$

(A5) $$\begin{array}{cc} \; & =\sum_{j=1}^{m}\frac{\partial }{\partial \theta_{i}}\left[x_{ij}\log\left(\lambda_{ij}\right)-\lambda_{ij}-\log\left(x_{ij}!\right)\right] \end{array}$$

(A6) $$\begin{array}{cc} \; & =\sum_{j=1}^{m}\frac{\partial }{\partial \theta_{i}}\left[x_{ij}\log\left(\lambda_{ij}\right)-\lambda_{ij}\right] \end{array}$$

(A7) $$\begin{array}{cc} \; & =\sum_{j=1}^{m}\frac{\partial }{\partial \theta_{i}}\left[x_{ij}\left(a_{j}\theta_{i}+b_{j}\right)-exp\left(a_{j}\theta_{i}+b_{j}\right)\right] \end{array}$$

(A8) $$\begin{array}{cc} \; & =\sum_{j=1}^{m}\left[x_{ij}a_{j}-a_{j}\exp\left(a_{j}\theta_{i}+b_{j}\right)\right] \end{array}$$

(A9) $$\begin{array}{cc} \; & =\sum_{j=1}^{m}a_{j}\left[x_{ij}-\exp\left(a_{j}\theta_{i}+b_{j}\right)\right]. \end{array}$$

### Appendix A.4. Second Derivative of the Log-Likelihood

By derivating the first derivative of the log-likelihood, we obtain the following:

(A10) $$\begin{array}{cc} \frac{\partial^{2}\ell \left(\lambda_{ij}\right)}{\partial \theta_{i}^{2}} & =\frac{\partial }{\partial \theta_{i}}\sum_{j=1}^{m}a_{j}\left[x_{ij}-\exp\left(a_{j}\theta_{i}+b_{j}\right)\right] \end{array}$$

(A11) $$\begin{array}{cc} \; & =\sum_{j=1}^{m}a_{j}\frac{\partial }{\partial \theta_{i}}\left[x_{ij}-\exp\left(a_{j}\theta_{i}+b_{j}\right)\right] \end{array}$$

(A12) $$\begin{array}{cc} \; & =\sum_{j=1}^{m}a_{j}\frac{\partial }{\partial \theta_{i}}\left[x_{ij}-\exp\left(a_{j}\theta_{i}+b_{j}\right)\right] \end{array}$$

(A13) $$\begin{array}{cc} \; & =\sum_{j=1}^{m}-a_{j}^{2}\exp\left(a_{j}\theta_{i}+b_{j}\right) \end{array}$$

(A14) $$\begin{array}{cc} \; & =-\sum_{j=1}^{m}a_{j}^{2}\lambda_{ij}. \end{array}$$

### Appendix A.5. Test Information

The second derivative of the log-likelihood can be used to obtain the test (or total) information function $I(\theta_{i})$, using the Poisson distribution property $E\left[\lambda_{ij}\right]=\lambda_{ij}$:

(A15) $$\begin{array}{cc} I(\theta_{i}) & =-E\left[\frac{\partial^{2}\ell \left(\theta_{i}\right)}{\partial \theta_{i}^{2}}\right] \end{array}$$

(A16) $$\begin{array}{cc} \; & =-E\left[-\sum_{j=1}^{m}a_{j}^{2}\lambda_{ij}\right] \end{array}$$

(A17) $$\begin{array}{cc} \; & =E\left[\sum_{j=1}^{m}a_{j}^{2}\lambda_{ij}\right] \end{array}$$

(A18) $$\begin{array}{cc} \; & =\sum_{j=1}^{m}E\left[a_{j}^{2}\lambda_{ij}\right] \end{array}$$

(A19) $$\begin{array}{cc} \; & =\sum_{j=1}^{m}a_{j}^{2}E\left[\lambda_{ij}\right] \end{array}$$

(A20) $$\begin{array}{cc} \; & =\sum_{j=1}^{m}a_{j}^{2}\lambda_{ij} \end{array}$$

(A21) $$\begin{array}{cc} \; & =\sum_{j=1}^{m}a_{j}^{2}e^{b_{j}+a_{j}\theta_{i}}, \end{array}$$

leading to Equation (7).

### Appendix A.6. Item Information

Similarly, the item information $I_{j}\left(\theta_{i}\right)$ can be obtained:

(A22) $$\begin{array}{cc} I_{j}\left(\theta_{i}\right) & =-E\left[\frac{\partial^{2}\ell_{j}\left(\lambda_{ij}\right)}{\partial \theta_{i}^{2}}\right] \end{array}$$

(A23) $$\begin{array}{cc} \; & =-E\left[-a_{j}^{2}\lambda_{ij}\right] \end{array}$$

(A24) $$\begin{array}{cc} \; & =I_{j}\left(\theta_{i}\right)=E\left[a_{j}^{2}\lambda_{ij}\right] \end{array}$$

(A25) $$\begin{array}{cc} \; & =a_{j}^{2}E\left[\lambda_{ij}\right] \end{array}$$

(A26) $$\begin{array}{cc} \; & =a_{j}^{2}\lambda_{ij} \end{array}$$

(A27) $$\begin{array}{cc} \; & =a_{j}^{2}e^{b_{j}+a_{j}\theta_{i}}, \end{array}$$

leading to Equation (8).
